# Motion-Preserving Salvage for Failed Total Ankle Arthroplasty or Talar AVN: Outcomes of Combined Total Ankle Arthroplasty with Custom 3D-Printed Total Talus Replacement

**DOI:** 10.1177/10711007261471856

**Published:** 2026-09-10

**Authors:** Rachit Saggar, Richard M. Smith, Eva Heidinger, Omar M. Yaldo, Carl T. Hasselman, MaCalus V. Hogan, Alex J. Kline

**Affiliations:** 1 Foot and Ankle Injury Research Group, Department of Orthopaedic Surgery, University of Pittsburgh, Pittsburgh, PA, USA; 2 Bethel Musculoskeletal Research Center [BMRC], Department of Orthopaedic Surgery, University of Pittsburgh School of Medicine, Pittsburgh, PA, USA; 3 The Centers for Advanced Orthopaedics, OrthoMaryland, Baltimore, MD, USA; 4 UHC Orthopaedics, Bridgeport, WV, USA

**Keywords:** total ankle arthroplasty, 3D-printed implant, total talus replacement, talar avascular necrosis, talar component collapse, subtalar arthrodesis

## Abstract

**Background:**

Talar component collapse following total ankle arthroplasty (TAA) and talar avascular necrosis (AVN) with concurrent ankle arthritis represent challenging clinical scenarios. Traditional salvage with tibiotalocalcaneal arthrodesis sacrifices motion and has nonunion rates approaching 30%. This study evaluates outcomes of a motion-preserving salvage strategy: combined TAA with custom 3D-printed total talus replacement (TAA-TTR).

**Methods:**

We retrospectively reviewed consecutive patients who underwent TAA-TTR between September 2020 and December 2023. Indications included talar component collapse following prior TAA or talar AVN with ankle and subtalar arthritis. Subtalar arthrodesis was performed using 2 screws with use of bone marrow aspirate concentrate. Primary endpoint was complication rate; secondary endpoints included reoperations, radiographic assessments, and functional recovery milestones.

**Results:**

Eighteen patients (10 females, 8 males; mean age 57.8 ± 10.8 years) were analyzed at mean follow-up of 36.8 ± 16.6 months, after excluding 2 with <12 months’ follow-up. Nine had talar AVN and 9 had failed TAA. The complication rate was 33.3% (95% CI: 15.2%-57.3%) (AVN group 44%, revision TAA group 22%). Six patients experienced complications: superficial surgical site infection (SSI) (1), deep SSI requiring debridement surgery (2), extensor hallucis longus rupture (1), nerve-related complication (1), and asymptomatic Chopart joint subluxation (1). Two patients required serial operations, including 1 below-knee amputation for persistent pain and infection. Reoperation rate was 11.1% (95% CI: 3.1%-32.8%). Patients achieved rapid functional recovery: controlled ankle motion boot transition at mean 19.9 days, full weight-bearing at mean 46.9 days, and regular footwear at mean 10.7 weeks. All patients achieved radiographic osseointegration of talar component to the calcaneus.

**Conclusion:**

TAA-TTR represents a viable motion-preserving procedure in this challenging population. Although patients achieved relatively rapid functional recovery and transition to regular footwear, rigorous selection remains key. This approach demonstrates a manageable complication rate while addressing tibiotalar and subtalar pathology simultaneously.

**Level of Evidence:**

Level IV, retrospective case series

## Introduction

End-stage ankle arthritis presents a significant challenge for foot and ankle surgeons. Current treatment options primarily include ankle arthrodesis or total ankle arthroplasty (TAA), each with their respective limitations. Arthrodesis eliminates joint motion and accelerates degeneration in adjacent joints due to increased biomechanical loads.^1^ Conventional TAA has become an increasingly viable alternative to fusion as it preserves joint motion, with recent long-term studies demonstrating significantly improved survival rates.^2^ Modern prosthetic designs now achieve 5-year survival rates of 85% to 88%, 10-year survival rates of 74% to 94%, and long-term survival extending to 76% at nearly 16 years.^3,4^ These data reflect substantial improvements in implant design, surgical technique, and patient selection when compared to earlier-generation devices.

Successful TAA relies on adequate bone stock at both the distal tibia and talar components for implant fit. Pre-existing or postoperative talar avascular necrosis or gradual talar component subsidence or collapse oftentimes compromise the stability of the implant with increased wear on the polyethylene liner as well as adjacent joints arthrosis. The management of talar collapse and AVN presents even greater challenges to TAA as these conditions traditionally require tibiotalocalcaneal (TTC) arthrodesis, resulting in functional limitations and loss of joint motion that patients wish to avoid. Furthermore, nonunion rates for TTC have been shown to approach 30%.^5^ For patients wishing to maintain ankle motion, options have been extremely limited.

Recent advances in 3D printing technology present a viable option, enabling the creation of custom talar prostheses that precisely match patient-specific anatomy.^6^ This technology permits a novel approach: total ankle arthroplasty with custom 3D-printed total talus replacement (TAA-TTR). This procedure offers several advantages: preservation of ankle motion, maintenance of limb length and alignment, complete resection of diseased talar bone, and treatment of subtalar arthritis.^7,8^

Subtalar arthrodesis is integral to the TAA-TTR procedure for several reasons. First, the 3D-printed talar component features a porous-coated undersurface specifically designed for osseointegration with the calcaneus rather than articulation. The combined procedure addresses both tibiotalar and subtalar pathology simultaneously, eliminating the need for staged procedures. Additionally, subtalar arthrodesis provides immediate structural stability during the osseointegration process.

Clinical data on this procedure remains limited. The TAA-TTR approach has been described for talar AVN, but only short-term outcomes have been reported.^6^ Its application and outcomes specifically for revision of failed TAA with talar component collapse has been minimally investigated. This study evaluates the clinical outcomes of patients undergoing TAA-TTR for 2 distinct salvage indications: talar component collapse after prior TAA and talar AVN with ankle arthritis. Although these represent fundamentally different patient populations with distinct risk profiles, both scenarios represent complex salvage situations. Our analysis focuses on recovery timeline, complication rates, and reoperation rates to determine whether TAA-TTR can serve as a viable salvage option in both clinical scenarios. We hypothesized that this treatment approach would yield acceptable complication rates, decreased reoperation rates compared with historical controls, and improved functional outcomes for these challenging cases.

## Materials and Methods

### Study Design and Patient Selection

This IRB-approved retrospective case series was conducted at a single academic institution. We retrospectively identified all patients who underwent total ankle arthroplasty with custom 3D-printed total talus replacement between September 2020 and December 2023. Indications included (1) talar component collapse following prior TAA (Figure 1) or (2) talar AVN with ankle and subtalar arthritis (Figure 2). Patients with less than 12 months follow-up were excluded from analysis.

**Figure 1.**
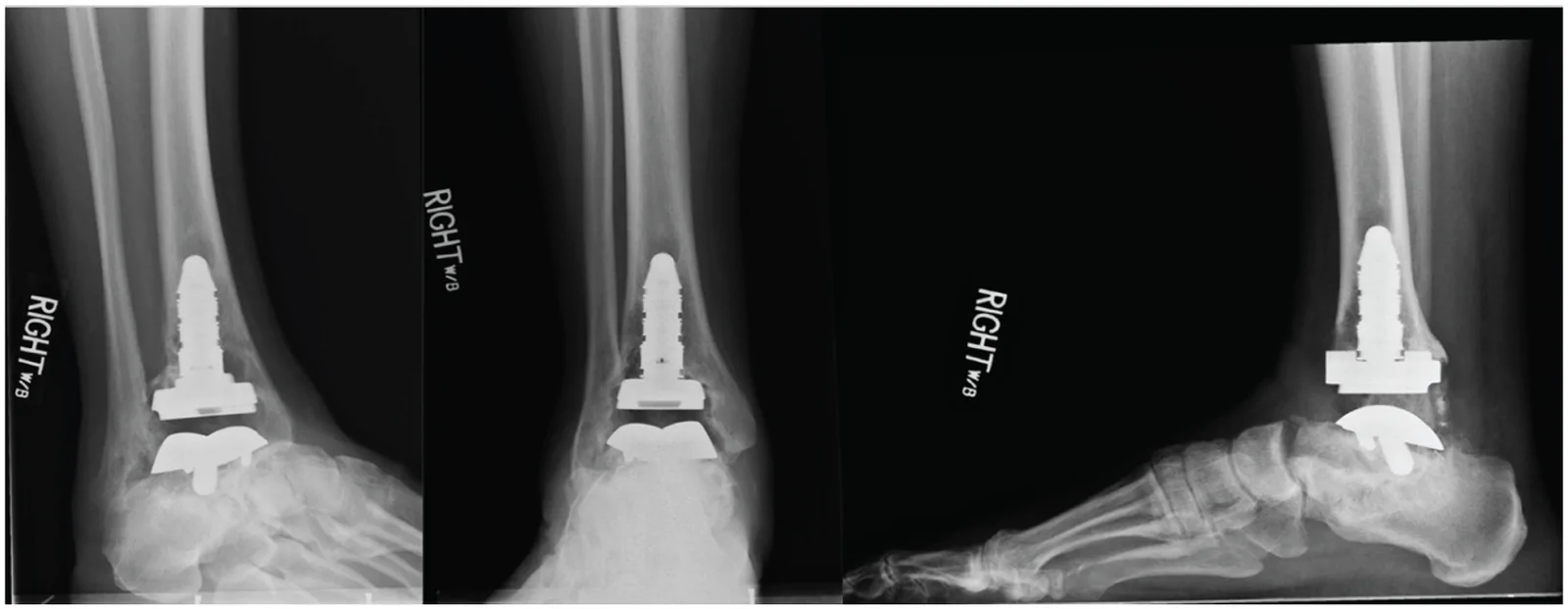
Preoperative mortise, anteroposterior, and lateral radiographs following a failed total ankle arthroplasty with significant talar component collapse (see Figure 5 for postoperative images).

**Figure 2.**
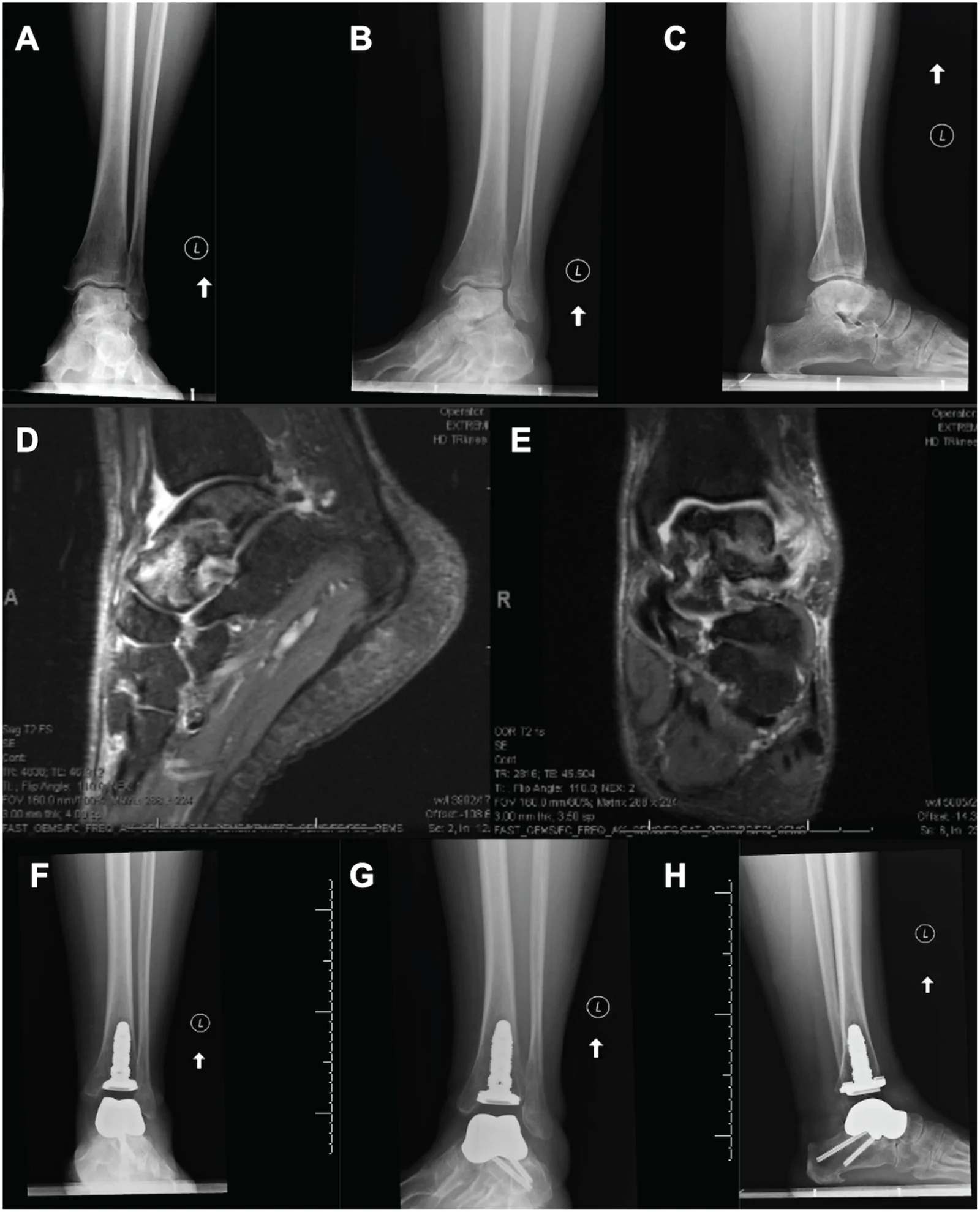
(A-C) Preoperative anteroposterior, mortise, and lateral radiographs of the left ankle demonstrating bony changes and sclerosis consistent with talar AVN. (D-E) Preoperative sagittal and coronal T2-weighted MRI sections of a patient with talar avascular necrosis. T2-weighted image demonstrates diffuse low signal intensity throughout the talar body with surrounding edema, consistent with extensive talar AVN. (E) Coronal image shows collapse of the talar dome with subchondral fracture and associated tibiotalar joint effusion. (F-H) Postoperative radiographs (3-month follow-up) of the left ankle following TAA-TTR demonstrate appropriate component alignment with an intact INBONE component and no evidence of hardware complication. AVN, avascular necrosis; MRI, magnetic resonance imaging; TAA-TTR, total ankle arthroplasty with custom 3D-printed total talus replacement.

The primary endpoint was complication rate, defined as any adverse event requiring intervention or causing significant clinical impact, excluding asymptomatic radiographic findings. Secondary endpoints included reoperation rate, radiographic assessments, functional recovery milestones (time to controlled ankle motion [CAM] boot, full weight-bearing, regular footwear), and patient-reported outcome measures.

### Preoperative Evaluation and Planning

All patients underwent standard preoperative assessment including history, physical examination, and weight-bearing radiographs. Advanced imaging included CT scans of both ankles. For talar collapse cases, contralateral ankle CT scans created mirror-image reconstructions.^9^ For AVN cases, viable talar bone was used in conjunction with contralateral imaging. Three-dimensional reconstructions were created by the implant manufacturer (Restor3D) and reviewed by the surgical team prior to implant production.

### Surgical Technique

Three fellowship-trained foot and ankle orthopaedic surgeons performed all procedures. Procedures were performed under general anesthesia with a regional nerve block, and a standard anterior approach to the ankle was used. In revision cases, failed components were removed. The talus was carefully resected en bloc or piecemeal. The distal tibia was prepared in standard fashion using either Wright Medical/Stryker Prophecy protocol or INBONE instrumentation.

The custom talar component sizing process combines patient-specific design with intraoperative flexibility. Preoperatively, CT scanning of the contralateral ankle creates a mirror-image 3D reconstruction of the patient’s native talar anatomy. The manufacturer (Restor3D) designed a patient-specific talar component based on this reconstruction and produces 3 size variants (small, medium, large) of the same patient-specific geometry while maintaining the custom anatomic shape. All 3 sterile custom implants are available in the operating room, along with corresponding trial components.

After talus resection and tibial preparation, trial components were used to assess fit and determine optimal sizing. The trial components were systematically tested for (1) appropriate articulation with the prepared tibial surface without point-loading or edge-loading, (2) proper seating on the calcaneal facet without overhang or gaps, (3) restoration of talocalcaneal height and hindfoot alignment, (4) adequate soft tissue tension without excessive tightness, and (5) appropriate ankle range of motion throughout dorsiflexion and plantarflexion. Fluoroscopic imaging confirmed proper component positioning and joint space recreation. Final size selection was based on the combination of tactile feedback, fluoroscopic assessment, and range of motion testing to ensure optimal fit without overstuffing or understuffing the joint space.

The subtalar joint was prepared for arthrodesis by denuding the articular cartilage and fenestrating the subchondral bone of the posterior and middle facets of the calcaneus. Care was taken to preserve the overall anatomic contour of the posterior facet to maintain appropriate talocalcaneal height and hindfoot alignment. The plasma-sprayed undersurface of the talar component was coated with bone marrow aspirate concentrate (BMAC) when available to enhance bony ingrowth. Subtalar fixation was achieved using two 6.5-mm screws in all cases. Fluoroscopy confirmed appropriate alignment, and range of motion testing verified stability.

### Postoperative Protocol

Patients were hospitalized postoperatively for observation for up to 24 hours. A splint was maintained for approximately 3 weeks before transition to a CAM boot with non-weight-bearing or toe-touch weight-bearing status. Progressive weight-bearing in the boot began at approximately 6 weeks, with transition to regular footwear and physical therapy between 10 and 12 weeks postoperatively. Postoperative radiographic assessment included weight-bearing anteroposterior, mortise, and lateral ankle radiographs obtained at 3 weeks, 6 weeks, 3 months, 6 months, and 12 months. In patients not yet cleared for full weight-bearing, simulated weight-bearing views were obtained using a radiolucent platform beneath the foot to apply axial load.

Successful subtalar integration was defined as stable incorporation of the porous-coated undersurface of the talar component with the calcaneus. This process represents osseointegration of a metal implant with host bone rather than traditional bone-to-bone arthrodesis. CT scans were obtained selectively when radiographic assessment was equivocal or when concern for loosening or incomplete integration existed.

### Data Collection and Analysis

We collected demographic data, medical history, surgical details, postoperative milestones, complications, and reoperations. Radiographic outcomes assessed component position, evidence of loosening, and subtalar fusion status. Patient-reported outcome measures were collected preoperatively and postoperatively and included visual analog scale (VAS) pain scores, Foot and Ankle Ability Measure Activities of Daily Living (FAAM ADL), and Patient-Reported Outcomes Measurement Information System (PROMIS) physical and mental health scores. Descriptive statistics were used to summarize demographic data, surgical characteristics, and outcomes. Continuous variables are presented as mean ± SD with ranges. Categorical variables are presented as frequencies and percentages. Ninety-five percent CIs were calculated for the outcomes where appropriate. Paired *t* tests were used to compare pre- and post-operative patient-reported outcome measures data. Normality of continuous variables was assessed using Shapiro-Wilk tests. However, given the limited sample size, the statistical power to detect departures from normality was inherently limited. Significance was set at *P* <.05.

## Results

### Demographics and Preoperative Status

Twenty consecutive patients underwent TAA-TTR between September 2020 and December 2023. Two patients with <12 months’ follow-up were excluded. The final cohort included 18 patients (10 females, 8 males) with mean age 57.8 ± 10.8 years (range, 36-78 years) and a mean BMI of 31.7 ± 5.5 (range, 19.6-42.9). The primary indications for surgery were talar component collapse following prior TAA (9 cases, 50%) and talar AVN with ankle arthritis (9 cases, 50%). Nine patients (50%) had undergone previous hindfoot or midfoot surgeries. Insurance status was evenly split between private insurance (9 patients) and Medicare (9 patients). Demographic characteristics and preoperative details appear in Table 1.

**Table 1.** Patient Demographics and Preoperative Characteristics.

Characteristic	Overall (n = 18)	Talar AVN (n = 9)	Revision TAA (n = 9)
Age, y, mean ± SD (range)	57.8 ± 10.8 (36-78)	54.6 ± 13.1 (36-78)	61.1 ± 7.2 (46-68)
Female, n (%)	10 (55.6)	5 (55.6)	5 (55.6)
BMI, mean ± SD	31.7 ± 5.5	32.2 ± 6.3	31.1 ± 5.0
Left laterality, n (%)	9 (50.0)	6 (66.7)	3 (33.3)
Previous hindfoot/midfoot surgery, n (%)	9 (50.0)	3 (33.3)	6 (66.7)
Diabetes, n (%)	1 (5.6)	1 (11.1)	0 (0.0)
Current tobacco use, n (%)	1 (5.6)	1 (11.1)	0 (0.0)
Former tobacco use, n (%)	10 (55.6)	4 (44.4)	6 (66.7)

Abbreviations: AVN, avascular necrosis; BMI, body mass index; TAA, total ankle arthroplasty.

### Surgical Details and Postoperative Outcomes

The mean clinical follow-up was 36.8 ± 16.6 months (range 12-57.6 months). The mean surgical duration was 124.6 minutes (range, 76-172 minutes). Total OR time averaged 156.1 minutes (range, 100-192 minutes). Total ankle replacement systems included INBONE II (Wright Medical) in 9 patients (50%) and Infinity (Wright Medical) in 9 patients (50%) (Figures 3 and 4). All patients remained hospitalized for at least 24 hours postoperatively. All subtalar arthrodeses achieved radiographic osseointegration, and no implants required removal for mechanical failure during the follow-up period.

**Figure 3.**
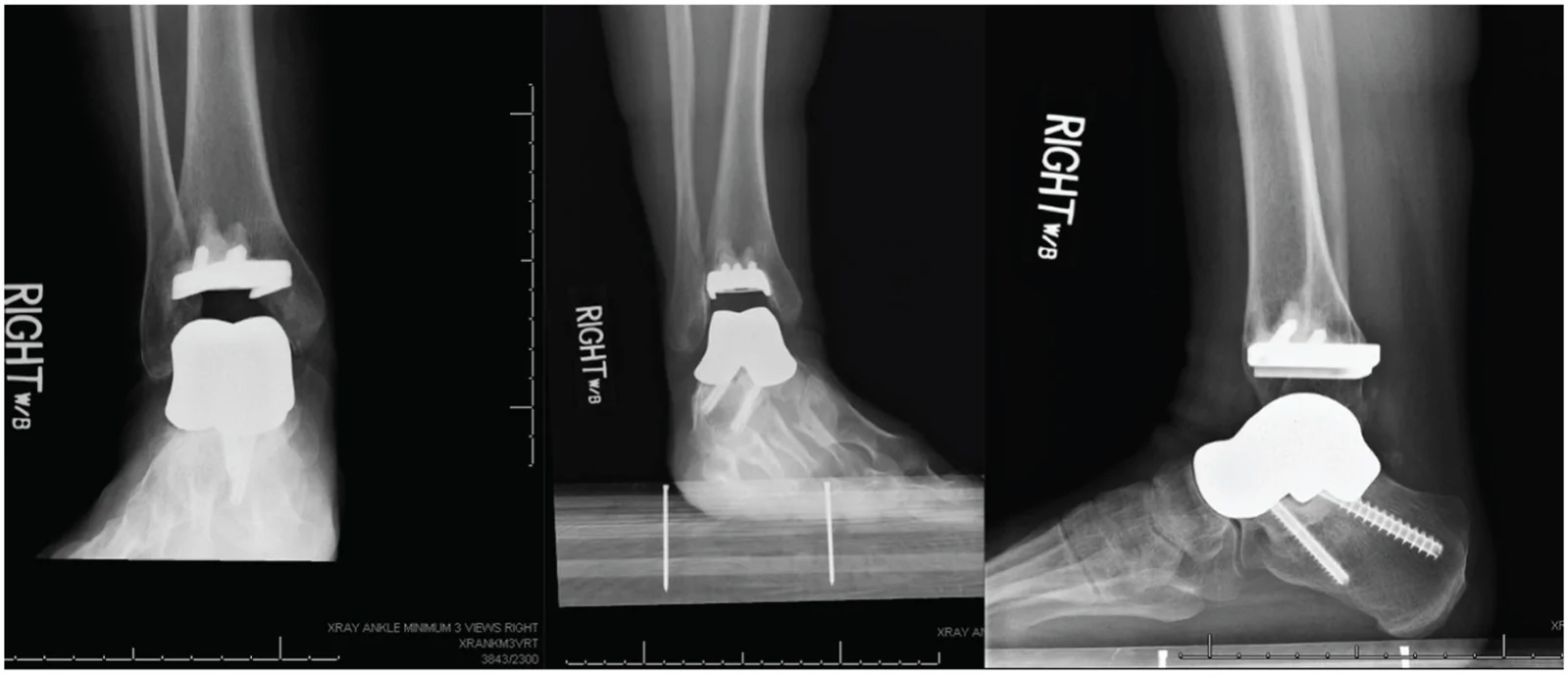
Postoperative (3-year and 25-day follow-up) anteroposterior, mortise, and lateral radiographs following total ankle arthroplasty with custom 3D-printed total talus replacement (TAA-TTR) for talar AVN with ankle arthritis.

**Figure 4.**
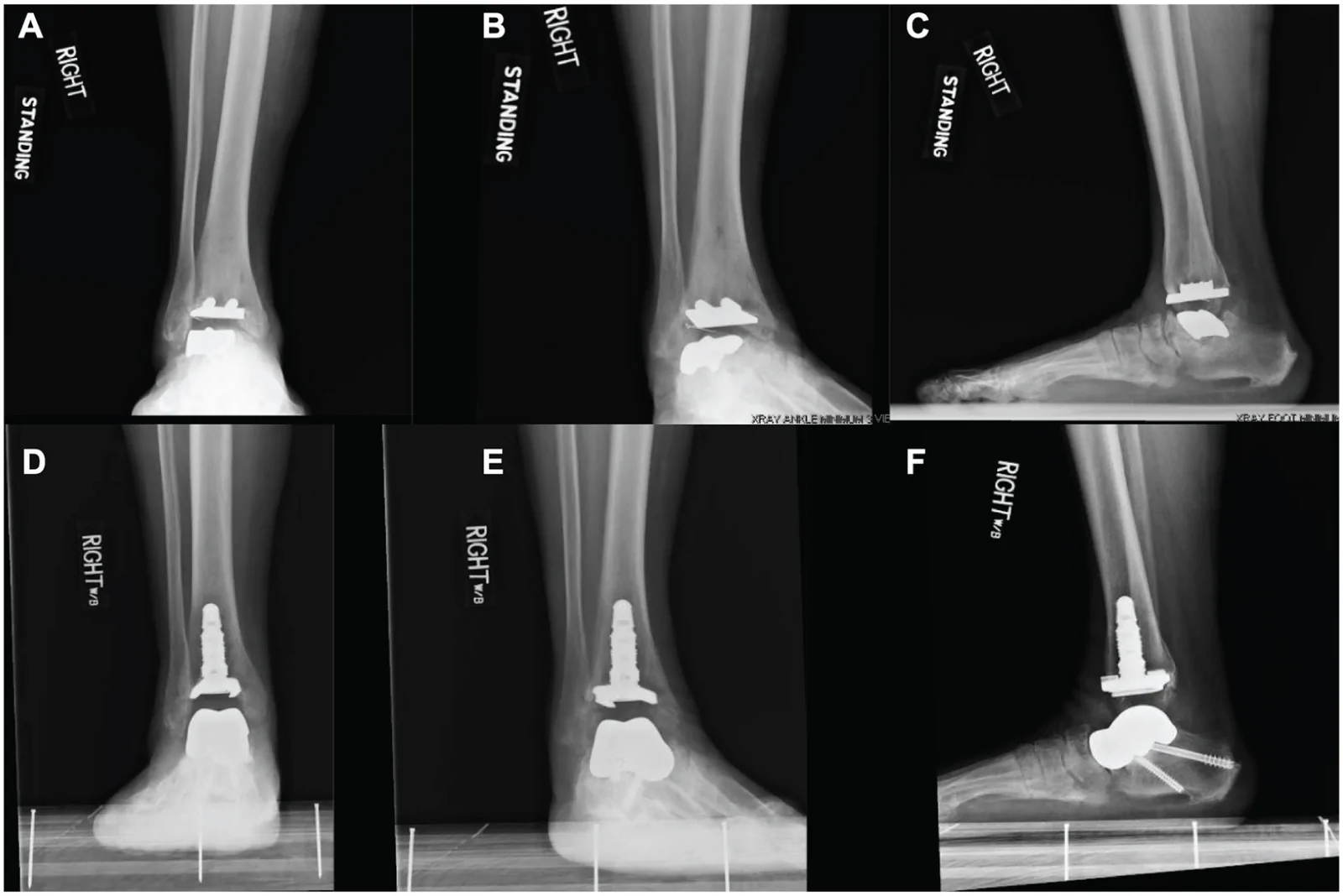
(A-C) Preoperative anteroposterior, mortise, and lateral radiographs demonstrating collapse of talar component. (D-F) Postoperative anteroposterior, mortise, and lateral radiographs following total ankle arthroplasty with custom 3D-printed total talus replacement (TAA-TTR) with INBONE II for prior talar component collapse.

Six patients developed complications (33.3%, 95% CI: 15.2%-57.3%), with 2 requiring reoperations (11.1%, 95% CI: 3.1%-32.8%). The complication rate was higher in the AVN group (4/9, 44.4%) compared with the revision TAA group (2/9, 22.2%). Notably, both patients who required reoperation had significant risk factors: patient 1 underwent below-knee amputation for persistent pain and infection was a former heavy smoker and a revision TAA case, whereas patient 2 required irrigation and debridement for deep infection, was a current smoker, and the only diabetic patient in the cohort. One patient developed progressive Chopart joint subluxation with diastasis at both the talonavicular and calcaneocuboid joints identified on surveillance radiographs (Figure 5). CT imaging confirmed stable talar component osseointegration without evidence of loosening or subsidence. The patient remains completely asymptomatic with no pain, instability symptoms, or functional limitations and has declined further intervention. Additional procedural details and clinical outcomes are presented in Table 2. Overall implant survivorship can be seen on the Kaplan-Meier curve in Figure 6 and was 88.9% (16/18) at 60 months.

**Figure 5.**
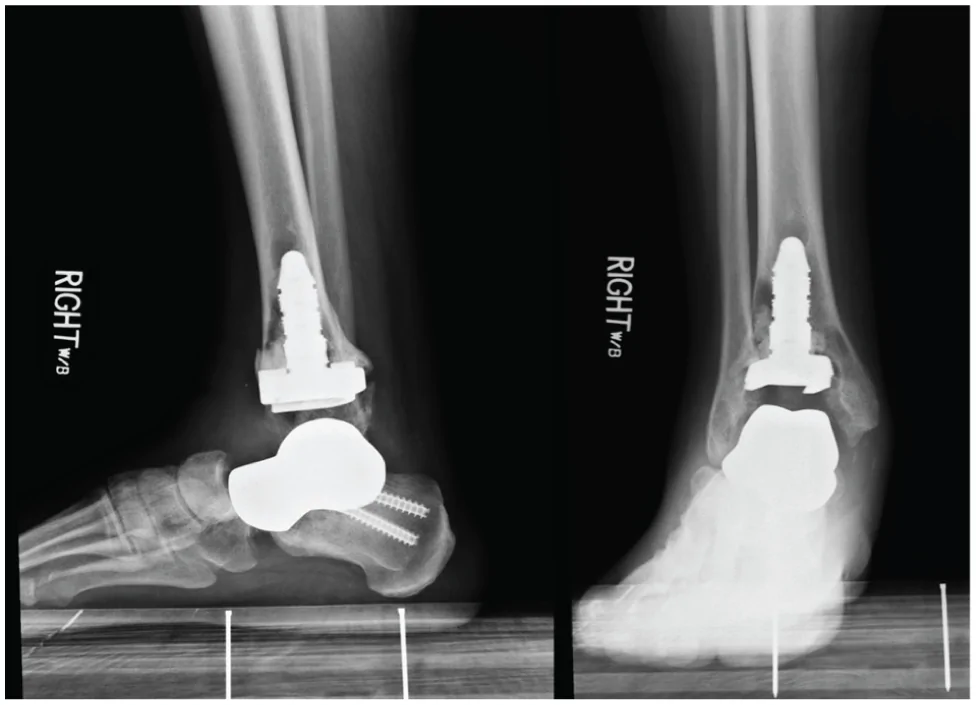
Postoperative (8-month follow-up) mortise and lateral radiographs depict progressive Chopart joint subluxation postoperatively in an asymptomatic patient (see Figure 1 for preoperative images).

**Table 2.** Surgical Details and Clinical Outcomes.

Parameter	Overall (n = 18)	Talar AVN (n = 9)	Revision TAA (n = 9)
Surgical details			
Surgical duration, min, mean (range)	124.6 (76-172)	115.8 (94-160)	132.6 (76-172)
Total OR time, min, mean (range)	156.1 (100-192)	150.0 (118-192)	161.6 (100-191)
Hospital stay duration, h, mean ± SD	31.3 ± 13.5	29.2 ± 9.4	33.4 ± 17.0
Concomitant procedures, n (%)			
BMAC harvest and application	16 (88.9)	8 (88.9)	8 (88.9)
Gastrocnemius recession (endoscopic)	4 (22.2)	2 (22.2)	2 (22.2)
Lateral ligament stabilization	4 (22.2)	2 (22.2)	2 (22.2)
Recovery milestones, mean (range)			
Transition to CAM boot, d	19.9 (13-22)	20.6 (19-21)	19.3 (13-22)
Full weight-bearing, d	46.9 (39-57)	47.3 (41-57)	46.7 (39-57)
Transition to regular shoes, wk	10.7 (7-12)	10.9 (9-12)	10.6 (7-12)
Clinical follow-up, mean ± SD			
Follow-up duration, mo	36.8 ± 16.6	42.9 ± 15.2	30.8 ± 16.5
Complications and reoperations, n (%)			
Complications	6 (33.3)	4 (44.4)	2 (22.2)
Reoperations	2 (11.1)	1 (11.1)	1 (11.1)

Abbreviations: AVN, avascular necrosis; BMAC, bone marrow aspirate concentrate; CAM, controlled ankle motion; TAA, total ankle arthroplasty.

**Figure 6.**
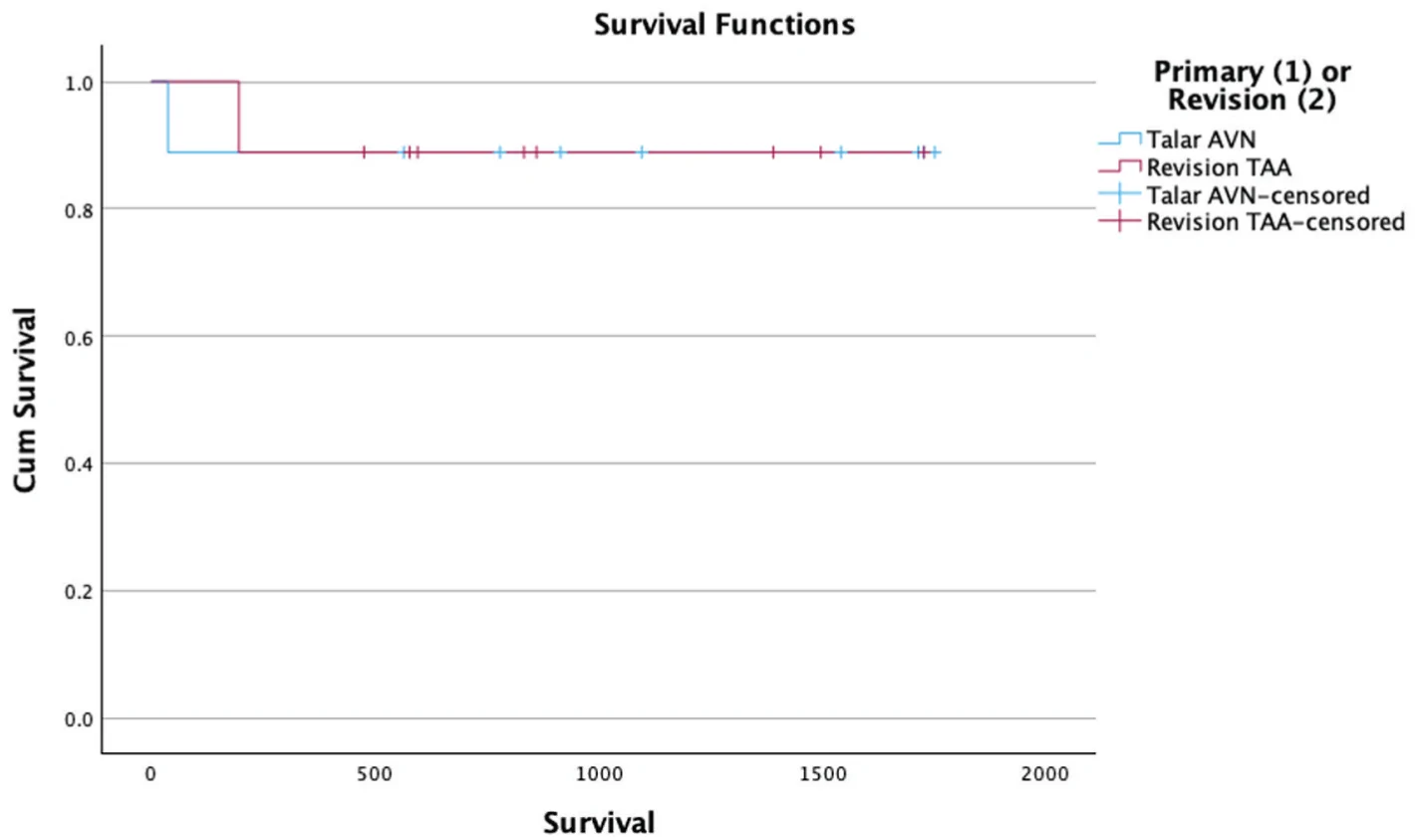
Kaplan-Meier survival curve comparing implant survival in total ankle arthroplasty with custom 3D-printed total talus replacement (TAA-TTR) patients with talar AVN vs revision TAA.

Recovery milestones demonstrated rapid functional recovery: patients transitioned to CAM boot at 19.9 days (95% CI: 19.0-20.8), achieved full weight-bearing at 46.9 days (95% CI: 44.2-49.7), and transitioned to regular footwear at 10.7 weeks (95% CI: 10.1-11.4). Recovery times were similar between indication groups.

Patient-reported outcome measures were collected preoperatively for all 18 patients and postoperatively for 16 patients (89% follow-up rate) at mean 12.8 ± 10.5 months, demonstrating statistically significant improvements in VAS pain score (*P* = .001), FAAM ADL score (*P* < .001), and PROMIS Physical Health score (*P* = .008); PROMIS Mental Health score did not reach statistical significance (*P* = .052) (Table 3).

**Table 3.** Patient-Reported Outcome Measures.

Outcome Measure	Pre-operative (n = 18)	Post-operative^a^ (n = 16)	*P* Value^b^
VAS pain score	5.7 ± 2.1	2.9 ± 2.1	**.001**
FAAM ADL score	41.1 ± 17.7	75.9 ± 11.9	**<.001**
PROMIS physical health	12.6 ± 1.5	14.0 ± 2.1	**.008**
PROMIS mental health	13.9 ± 2.5	15.1 ± 2.0	.052

a Post-operative scores were collected at mean 12.8 ± 10.5 months after surgery.

b Boldface indicates significance (*P* < .05).

Abbreviations: FAAM ADL, Foot and Ankle Ability Measure Activities of Daily Living; PROMIS, Patient-Reported Outcomes Measurement Information System; VAS, visual analog scale.

## Discussion

This study presents one of the larger series evaluating TAA-TTR in patients with complex talar pathology, including both failed TAA and talar AVN. With a mean follow-up of nearly 37 months, our findings provide critical insight into the clinical durability, recovery trajectory, and complication profile of this motion-preserving salvage procedure.

Talar collapse following TAA or extensive talar AVN represents one of the most challenging scenarios in foot and ankle surgery, particularly in patients seeking to maintain active lifestyles. Traditional salvage procedures, such as TTC arthrodeses, invariably sacrifice ankle and subtalar motion and are associated with significant functional limitations. Salvage arthrodesis following failed TAA has demonstrated fusion rates as low as 81%, with nonunion rates of approximately 10.6% and reoperation rates of 18%.^10^ Moreover, additional subtalar fusion yields fusion rates of only 61% following prior ankle arthrodesis (vs 92% with primary TAA), whereas alternative methods like posterior-plate TTC fusion and TTC nailing (particularly in diabetic patients) exhibit nonunion and compromised ambulation despite respectable limb salvage rates.^11-13^

Our reoperation rate of 11.1% compares favorably with these historical benchmarks, particularly considering that 50% of our cohort had undergone previous hindfoot or midfoot surgeries, factors known to complicate subsequent interventions and increase complication risk. The 100% subtalar osseointegration rate achieved in our series also contrasts with the 61% rate reported for subtalar fusion following ankle arthrodesis, suggesting potential biological advantages to the TAA-TTR approach.^11^

The development of talar replacement technology has progressed substantially over the past decade. Early-generation stainless steel talar body implants with peg fixation maintained integrity for 10-36 years, although 15% required revision for loosening or collapse.^14^ First-generation ceramic talar body devices produced only 54% good to excellent outcomes at 8 years, ultimately giving way to full total talus replacement designs. Modern studies reflect significant improvements: Kadakia et al^15^ reported significant improvements in VAS pain and FAOS scores with 100% survivorship at mid-term follow-up (with a reoperation rate of 11%). Similarly, Abar et al^16^ and Morita et al^14^ have demonstrated promising functional improvements and long-term implant survivorship.

The TAA-TTR approach has shown encouraging results across multiple studies. So et al^17^ demonstrated no significant differences in VAS or AOFAS scores between isolated total talus replacement and TAA-TTR procedures, with overall reoperation rates of 16.7% and survivorship of 94.5%. Wang et al^18^ reported significant improvements in PROMIS pain and function scores at 38 months with TAA-TTR, with a reoperation rate of 11%, whereas Madi et al^19^ documented improvements in VAS scores from 7.9 to 2.8 and a 100% subtalar/talonavicular fusion rate in a smaller TAA-TTR cohort.

Modern 3D-printed custom implants offer 3 distinct advantages over their predecessors: (1) patient-specific anatomic implants through mirror-image reconstruction of contralateral anatomy, (2) optimal biomechanical properties from titanium or cobalt-chrome alloys with porous-coated surfaces, and (3) integrated design addressing both tibiotalar and subtalar articulations. Lastly, the custom talar implant can be preoperatively modified to allow for additional porous coated articulation and screw fixation sites for talonavicular arthrodesis, if indicated.

By precisely replicating patient-specific talar anatomy, these custom implants enhance biomechanical function and minimize edge-loading. The integrated design, with a superior surface articulating with the tibial component and an inferior porous-coated surface optimized for subtalar osseointegration, addresses the frequent coexistence of subtalar arthritis, obviating the need for a separate fusion procedure. Radiographically, these devices restore talocalcaneal height and declination angle toward physiologic norms, whereas long-term studies with alumina ceramic implants have shown no subsidence.^14,20,21^ Modern designs also incorporate suture eyelets or screw-holes that permit optional subtalar fusion or ligament re-attachment, enhancing surgical versatility.

Recent studies support our findings on the benefits of TAA-TTR. Pereira et al^20^ demonstrated that this approach achieves high fusion rates in patients with end-stage talar osteonecrosis. Similarly, West and Rush^22^ and Hussain^23^ reported that modern metallic 3D-printed implants provide excellent biomechanical stability and promote strong integration with host bone; these observations are further supported by Jennison et al,^24^ who noted overall improved clinical performance with these devices.

One of the most clinically relevant findings in our series was the relatively rapid functional recovery timeline. Patients transitioned to a CAM boot at approximately 19.9 days, achieved full weight-bearing by 46.9 days, and resumed regular footwear by 10.7 weeks postoperatively. These recovery milestones are notably accelerated compared with the traditional 3-6-month timeline typically reported for TTC arthrodesis, representing a significant advantage in terms of patient quality of life and return to activities.^19,13^

The preservation of tibiotalar motion has been associated with reduced risk of adjacent joint arthritis, as Dekker et al^11^ have shown. So et al^17^ have demonstrated that TAA-TTR can achieve outcomes comparable to isolated total talus replacement while effectively addressing subtalar pathology.

Technical aspects, including soft tissue management and the subtalar fusion technique, are essential to achieving reliable outcomes. In our series, 4 patients underwent concurrent Brostrom stabilization with suture augmentation to maintain coronal plane stability, whereas the use of two 6.5-mm screws for subtalar arthrodesis provided excellent compression. In addition, careful selection between ankle prosthesis systems—using the INBONE system for cases with compromised tibial bone stock vs the Infinity system in primary cases—ensured that each patient’s unique anatomy and bone quality were properly addressed. These factors with the adjunctive use of BMAC were associated with the exceptional osseointegration rates and stability observed.

Patient selection remains the cornerstone of successful TAA-TTR outcomes. Ideal candidates demonstrate adequate bone stock, sufficient soft tissue quality, preserved vascular status, and realistic expectations. The 2 patients requiring reoperation had significant risk profiles: the patient who underwent below-knee amputation was a former heavy smoker with a revision TAA, whereas the patient requiring I&D was an active smoker with diabetes mellitus. These cases highlight the importance of comprehensive preoperative risk stratification. Although our cohort spanned ages 36-78 years, physiologic age rather than chronologic age should guide decision making. Our experience indicates that modifiable risk factors, particularly tobacco use, diabetes, and prior surgical interventions, may be more predictive of complications than BMI or age alone. Recent research identifying depression as a strong predictor of secondary surgery suggests potential benefit from psychological screening during patient selection.^21^

### Limitations

Several limitations must be acknowledged. First, this is a retrospective case series without a control group, making it impossible to directly compare outcomes with alternative treatment approaches. Second, standardized patient-reported outcome measures were not consistently available across all patients, limiting our ability to quantitatively assess functional improvement. Third, the sample size (n = 18) is relatively small, though comparable to existing literature on this specialized procedure. Fourth, the mean follow-up still represents short-term rather than long-term results. Additionally, as a single institution with considerable expertise in complex ankle reconstruction, generalizability may be limited. Although we included patients with 2 distinct indications, this heterogeneity reflects the clinical reality that TAA-TTR serves as a salvage option for multiple pathologies. With only 9 patients in each group, we lack statistical power to make definitive comparisons between indications or to recommend one approach over established alternatives. Our intent is to present preliminary outcomes of this novel technique, and the results should be interpreted as such, warranting future comparative studies with larger sample sizes.

## Conclusion

Total ankle arthroplasty with custom 3D-printed total talus replacement represents a promising approach for managing complex ankle pathology. The results demonstrate acceptable complication and reoperation rates, suggesting this procedure may be a viable alternative to TTC arthrodesis in appropriately selected patients. The combined approach addresses both ankle and subtalar joint pathology while preserving ankle motion. Notably, patients achieved relatively rapid functional recovery and transition to regular footwear. This represents a significant functional advantage compared with traditional salvage approaches. The 100% subtalar osseointegration rate and absence of implant failures during follow-up suggest that with appropriate patient selection and meticulous technique, this procedure can provide reliable outcomes for these challenging cases.

## Supplemental Material

sj-pdf-1-fai-10.1177_10711007261471856 – Supplemental material for Motion-Preserving Salvage for Failed Total Ankle Arthroplasty or Talar AVN: Outcomes of Combined Total Ankle Arthroplasty with Custom 3D-Printed Total Talus Replacement Supplemental material, sj-pdf-1-fai-10.1177_10711007261471856 for Motion-Preserving Salvage for Failed Total Ankle Arthroplasty or Talar AVN: Outcomes of Combined Total Ankle Arthroplasty with Custom 3D-Printed Total Talus Replacement by Rachit Saggar, Richard M. Smith, Eva Heidinger, Omar M. Yaldo, Carl T. Hasselman, MaCalus V. Hogan and Alex J. Kline in Foot & Ankle International

## Data Availability

The datasets generated during and/or analyzed during the current study are available from the corresponding author on reasonable request.
